# Surgical accuracy of open platform image-based robotic-assisted total knee arthroplasty across different implants: a multicentre trial

**DOI:** 10.1186/s42836-025-00334-x

**Published:** 2025-10-07

**Authors:** Michael Tim-Yun Ong, Chuan He, Wei Chai, Rex Wang-Fung Mak, Cham-Kit Wong, Gloria Yan-Ting Lam, Tsz Lung Choi, Patrick Shu-Hang Yung

**Affiliations:** 1 Chinese University of Hong Kong, Hong Kong SAR, China; 2 CUHK Medical Centre, Hong Kong SAR, China; 3 Ruijin Hospital, Shanghai Jiao Tong University School of Medicine, Shanghai, 200025 China; 4 Fourth Medical Centre of Chinese PLA General Hospital, Beijing, 100089 China; 5 Prince of Wales Hospital, Hong Kong SAR, China; 6 Alice Ho Miu Ling Nethersole Hospital, Hong Kong SAR, China

**Keywords:** Robotic-assisted total knee arthroplasty, Open-platform total knee arthroplasty, Total knee arthroplasty, Implant alignment, Radiological accuracy

## Abstract

**Background:**

Implant malalignment in total knee arthroplasty (TKA) correlates with poor outcomes, and robotic-assisted systems aim to improve precision. While closed-platform robotic systems dominate the market, their restriction to proprietary implants limits surgical flexibility. This study evaluates the radiological accuracy of an open-platform robotic system (Yuanhua KUNWU) across four TKA implant designs.

**Methods:**

A multi-centre retrospective analysis of 129 robotic-assisted TKAs (Zhengtian Irene, *n* = 60; DePuy Synthes Attune, *n* = 32; Zimmer Biomet Persona, *n* = 20; Smith & Nephew Legion, *n* = 17) was conducted. Patients with end-stage osteoarthritis (Kellgren-Lawrence grade 3–4) were included, while those with prior knee surgery or complex anatomy were excluded (*n* = 15). A total of 114 pre-operative and post-operative alignment (hip-knee-ankle angle [HKA], femoral and tibial component coronal angles [FCCA, TCCA], posterior tibial slope [PTS]) were measured on radiographs by two independent reviewers. Interobserver reliability (intra-class correlation [ICC], Cronbach’s α) and deviations from planned alignment (paired *t*-tests) were analysed. Acceptability was defined as ≤ 3° deviation.

**Results:**

Interobserver reliability was excellent (ICC > 0.77, Cronbach’s α > 0.87 for all parameters). Mean post-operative deviations from planned alignment were clinically small: HKA (+1.32°, *P* < 0.001), FCCA (−0.55°, *P* < 0.001), TCCA (+0.19°, *P* = 0.097), and PTS (−0.42°, *P* = 0.018). All mean differences were within the 3° acceptability threshold. Subgroup analysis of pre- and post-operative alignment between implant types also showed deviations of < 3°.

**Conclusions:**

The KUNWU open-platform robotic system achieved high radiological accuracy across four implant designs, with alignment deviations < 1.5°. This suggests open-platform robotics can provide implant versatility without compromising precision. Further studies regarding the assessment of long-term clinical and patient-reported outcomes and comparison with closed-platform systems are warranted.

## Introduction

Total knee arthroplasty (TKA) remains one of the most successful and cost-effective interventions for end-stage knee osteoarthritis, offering significant pain relief and functional improvement for the majority of patients [1]. However, despite advancements in surgical techniques, implant design, and perioperative care, patient dissatisfaction persists in 15–25% of cases, often attributed to residual pain, stiffness, or perceived instability [2, 3]. Implant malalignment has been suggested to cause poor patient-reported outcome measures (PROMs) [4] as well as decreased implant survival, in particular, aseptic loosening [5, 6]. In particular, more than 3° deviation from the mechanical axis has been suggested to be associated with implant loosening [7]. Some other studies, however, claim no difference in outcome or survivorship in mechanical axis deviation [8]. Nonetheless, accurate execution of the surgical plan and placement of the implant are still of importance to achieve the surgeon’s goal.

To enhance surgical precision, computer-assisted and robotic-assisted TKA systems have been developed, offering improved accuracy in bone resection and component positioning compared to conventional techniques [9–11]. Robotic systems provide an additional benefit of providing haptic feedback, which limits the risk of peri-articular soft tissue and bone trauma. Studies have also shown that robotic systems have improved accuracy, short-term outcomes, and earlier recovery in comparison with computer navigation [12, 13]. Robotic systems can be broadly classified into closed- and open-platform designs. Closed-platform systems, which dominate the current market, are proprietary and restrict surgeons to a single manufacturer’s implants, limiting flexibility in implant selection and alignment strategies [14]. While these systems benefit from implant-specific biomechanical optimization, their lack of cross-compatibility may not accommodate variations in patient anatomy or surgeon preference.

In contrast, open-platform robotic systems allow for the use of multiple implant brands and designs, enabling surgeons to tailor prosthesis selection based on individual patient factors, such as bone morphology, ligamentous stability, and kinematic requirements. This flexibility is particularly advantageous in complex cases where a one-size-fits-all approach may be suboptimal. However, concerns remain regarding whether open-platform systems can achieve comparable alignment accuracy given the absence of implant-specific algorithms that are integral to closed-platform systems [15].

The Yuanhua’s KUNWU system represents a novel CT-based open-platform semi-active robotic system. Bone cuts are performed using a navigated blade with haptic boundaries, minimising injury to surrounding tissues. Its open-platform system permits the use of multiple TKA implant systems as well as 3D-printed patient-specific implants. While early studies on robotic TKA have demonstrated improved alignment precision, most have focused on closed-platform systems, leaving a gap in the literature regarding the reliability of open-platform alternatives [16, 17]. This multi-centre retrospective study evaluates the radiological accuracy of the KUNWU system across four TKA implants (DePuy Synthes Attune, Smith & Nephew Legion, Zhengtian Irene, and Zimmer Biomet Persona), assessing its ability to translate preoperative plans into accurate postoperative alignment.

## Methods

This is a retrospective analysis of prospectively collected data in the authors’ institutional joint registry. Ethics approval was obtained from the Institutional Ethics Review Committee of each respective hospital.

PASS 15 (NCSS LLC, Kaysville, USA) was used to estimate the sample size based on paired sample *t*-tests. Assuming a significance level of 0.05, power of 0.80, and a conservative effect size of 0.3 (small-to-medium), the sample size was calculated to be 90.

**Fig. 1:**
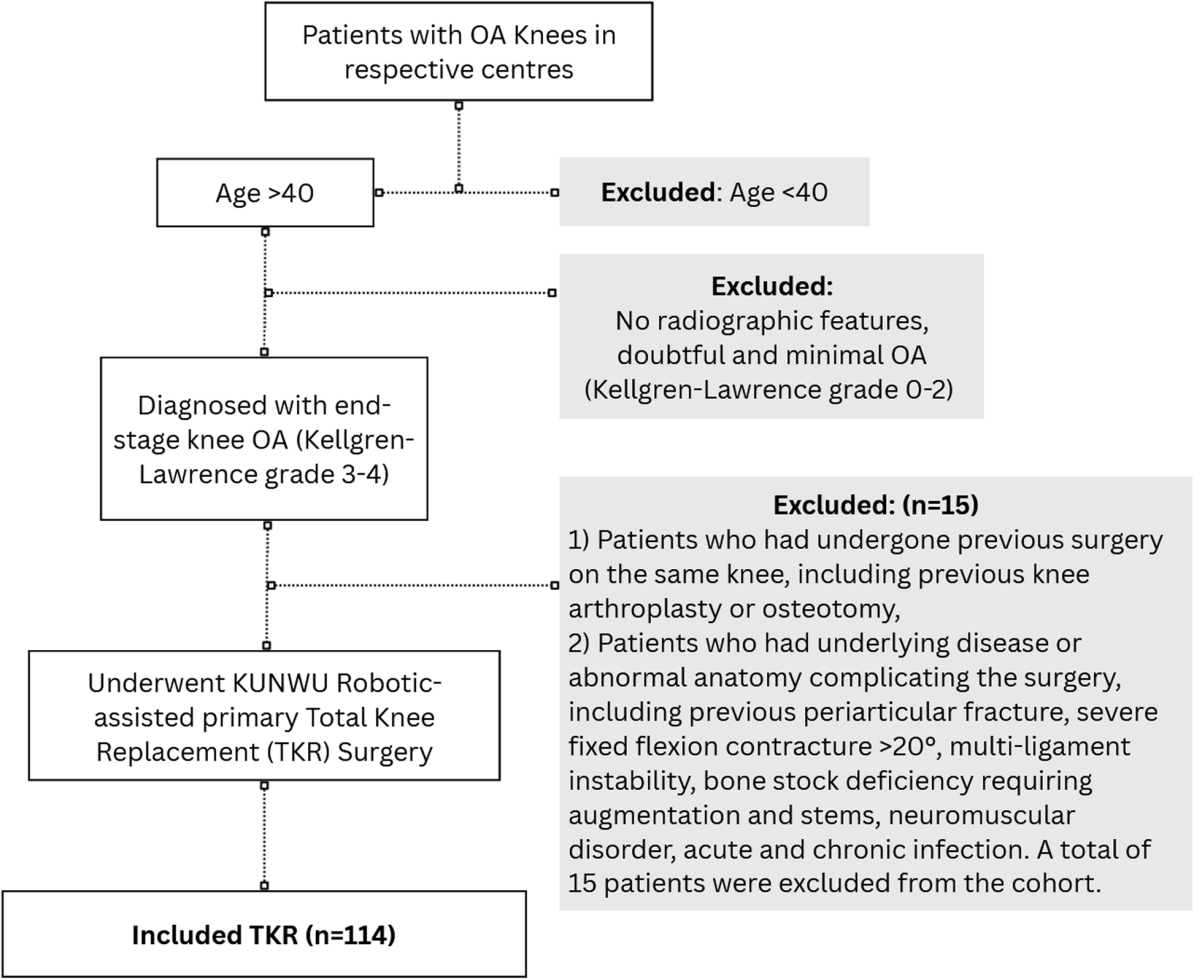
Inclusion and exclusion criteria flowchart

A total of 129 patients over 40 years old who suffered from end-stage osteoarthritis of the knee (Kellgren and Lawrence grade 3–4) who underwent KUNWU robotic-assisted primary total knee replacement were retrospectively examined. Exclusion criteria were 1) all patients who had undergone previous surgery on the same knee, including previous knee arthroplasty or osteotomy, 2) all patients who had underlying disease or abnormal anatomy complicating the surgery, including previous periarticular fracture, severe fixed flexion contracture > 20°, multi-ligament instability, bone stock deficiency requiring augmentation and stems, neuromuscular disorder, acute and chronic infection. A total of 15 patients were excluded from the cohort. (Fig. 1).

**Fig. 2:**
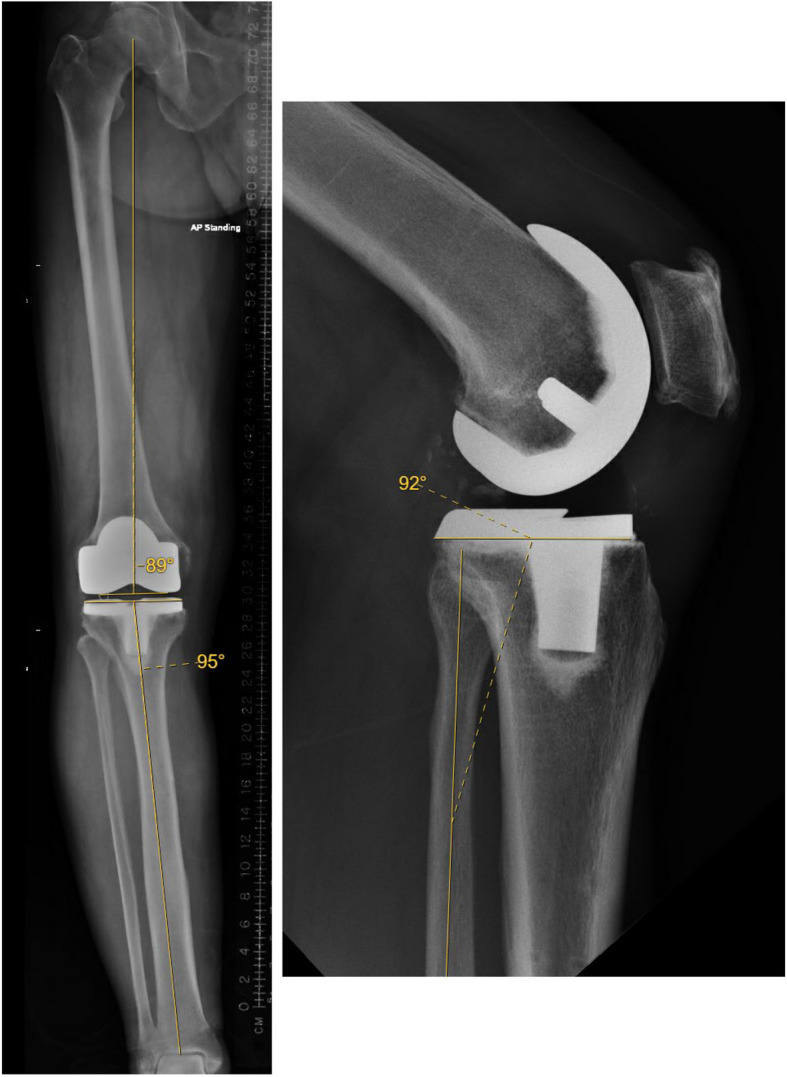
Left: Measurement of Femoral Component Coronal Alignment (FCCA) and Tibial Component Coronal Alignment (TCCA). Right: Posterior Tibial Slope (PTS) using fibular shaft axis as reference

All total knee arthroplasties were performed using functional or anatomic alignment as per the surgeons’ preferences. Choice of implant was based on the surgeon’s preference out of all the implants available to the open-platform robotic system. There was a total of 6 surgeons, who were all knee specialists or fellowship-trained total joint arthroplasty surgeons, and all are high-volume surgeons as defined in the literature [18, 19]. Clinical data included the patient's demographic data, operation records, and radiographs. The preoperative and postoperative radiographs were reviewed by two independent reviewers, and any discrepancies were reviewed and verified by the principal investigator. These included full-length standing anterior to posterior hip to ankle radiographs and short-standing anterior–posterior and lateral knee radiographs. Measurements performed included pre-operative, planned, and post-operative hip-knee-ankle angle (HKA), femoral component coronal alignment (FCCA), tibial component coronal alignment (TCCA), and posterior tibial slope (PTS) (Fig. 2). FCCA and TCCA were defined as the angle between the long axis of the component and the femoral and tibial mechanical axis, respectively. PTS was measured against the fibular shaft axis (Fig. 2). Interobserver reliability was evaluated by comparing the radiological measurements on the same set of radiographs between two independent observers. The outcome was defined as acceptable when the values were within 3° and as outliers when the values deviated from the planned angle by more than 3°.

Planned and Post-operative HKA, femoral component coronal angle, tibial component coronal angle, and posterior tibial slope were compared between the two groups using a paired *t*-test. Further subgroup analyses were performed to look at the difference between pre- and post-operative alignment between functional alignment (FA) and mechanical alignment (MA), as well as between implant types. Inter-rater reliability was used to compare radiological measurements between two independent orthopaedic surgeons through intra-class correlation (ICC) and Cronbach’s *α* values. All statistical analyses were done using IBM SPSS version 28 (Armonk, NY: IBM Corp). Statistical significance was set at *P* < 0.05.

## Results

There was a total of 114 knees with TKAs performed using 4 different implants (59 ZhengTian Irene, 25 DePuy Synthes Attune, 15 Zimmer Biomet Persona, 15 Smith & Nephew Legion). Thirty-four percent of patients were male, with a mean age of 66.8 years (range 53–82, 1SD 6.19) (Table 1). There were no untoward complications, including fracture, neurovascular injury, or venous thromboembolism. Post-operative radiographs were obtained at least 4–6 weeks from index surgery, especially the full-length hip-to-ankle radiographs. This was done to optimise the knee range of motion and prevent recurrence of post-operative flexion contracture, which would lead to inaccurate measurement of HKA and implant position.

**Table 1:** Patient demographics

Gender	
Female	85 (65.38%)
Male	45 (34.63%)
Age	
Mean	66.82
Std. Deviation	6.19
Minimum	53
Maximum	82
95% Confidence Interval of Mean	65.75–67.9

The interobserver reliability analysis demonstrated excellent agreement between the two raters across all measured parameters (Table 2). The Hip-Knee-Ankle (HKA) angle showed near-perfect reliability, with a Cronbach’s alpha of 0.986 and an ICC of 0.972 (95% CI: 0.959–0.981). Similarly, the Femoral Component Coronal Alignment (FCCA) and Tibial Component Coronal Alignment (TCCA) angles exhibited strong reliability, with alpha values of 0.936 and 0.911, and ICCs of 0.877 (95% CI: 0.824–0.914) and 0.836 (95% CI: 0.770–0.885), respectively. Posterior Tibial Slope (PTS) angle also showed good agreement (α = 0.873, ICC = 0.776, 95% CI: 0.694–0.843). Overall, the results indicate highly consistent measurements between raters, with HKA being the most reliable parameter.

**Table 2:** Interobserver Reliability between two raters

Measurement	Cronbach α	ICC	95%CI lower	95%CI upper
HKA	0.986	0.972	0.959	0.981
FCCA	0.936	0.877	0.824	0.914
TCCA	0.911	0.836	0.770	0.885
PTS	0.873	0.776	0.694	0.843

Paired *t*-test results revealed statistically significant differences between pre-operative (planned) and post-operative measurements for three out of four parameters (Table 3). FCCA showed a small but significant decrease post-operatively (mean difference: −0.55°, *P* < 0.001), while TCCA showed no significant change (mean difference: +0.19°, *P* = 0.097). The PTS angle exhibited a marginal post-operative reduction (mean difference: −0.42°, *P* = 0.018). The Hip-Knee-Ankle (HKA) angle showed a statistically significant planned-to-post-operative change (+1.32°, *P* < 0.001). However, the numerical value of the mean difference as well as the standard deviation ranges were still below the threshold of 3°, which was deemed a radiologically acceptable outcome.

**Table 3:** Paired *t*-test results between pre-operative and post-operative measurements of the whole cohort

Measurement	Planned (SD)	Post-op (SD)	Difference (SD)	95%CI lower	95%CI upper	*P*-value
HKA	179.49 (1.11)	178.17 (1.31)	1.32 (1.04)	1.13	1.52	0.000
FCCA	90.18 (0.63)	90.73 (1.53)	− 0.55 (1.41)	− 0.81	− 0.29	0.000
TCCA	89.72 (0.78)	89.53 (1.36)	0.19 (1.19)	− 0.03	0.41	0.097
PTS	86.74 (0.62)	87.16 (2.04)	− 0.42 (1.85)	− 0.76	− 0.07	0.018

**Fig. 3:**
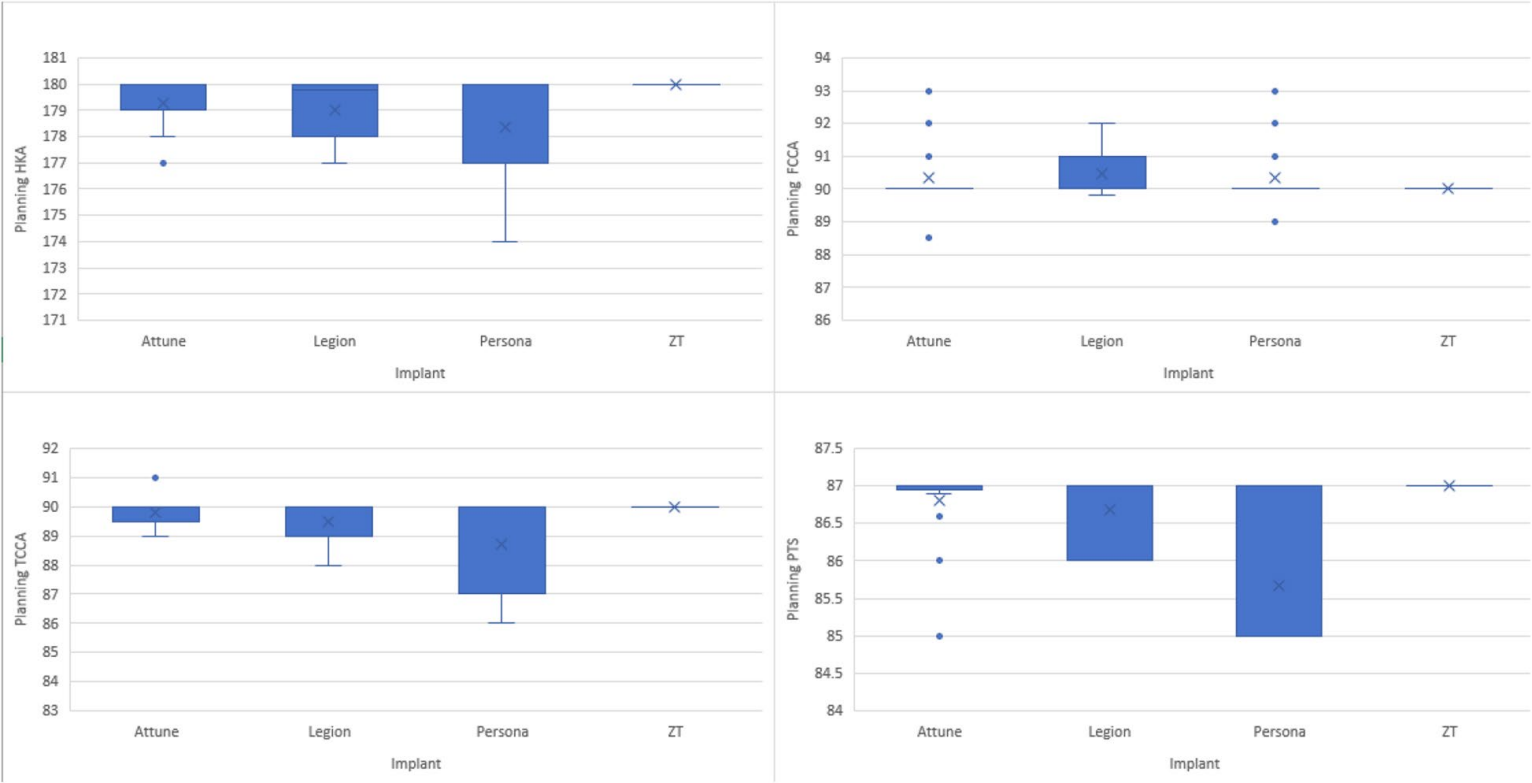
Box graphs of pre-operative alignment planning of HKA, FCCA, TCCA, and PTS divided by implant types

**Fig. 4:**
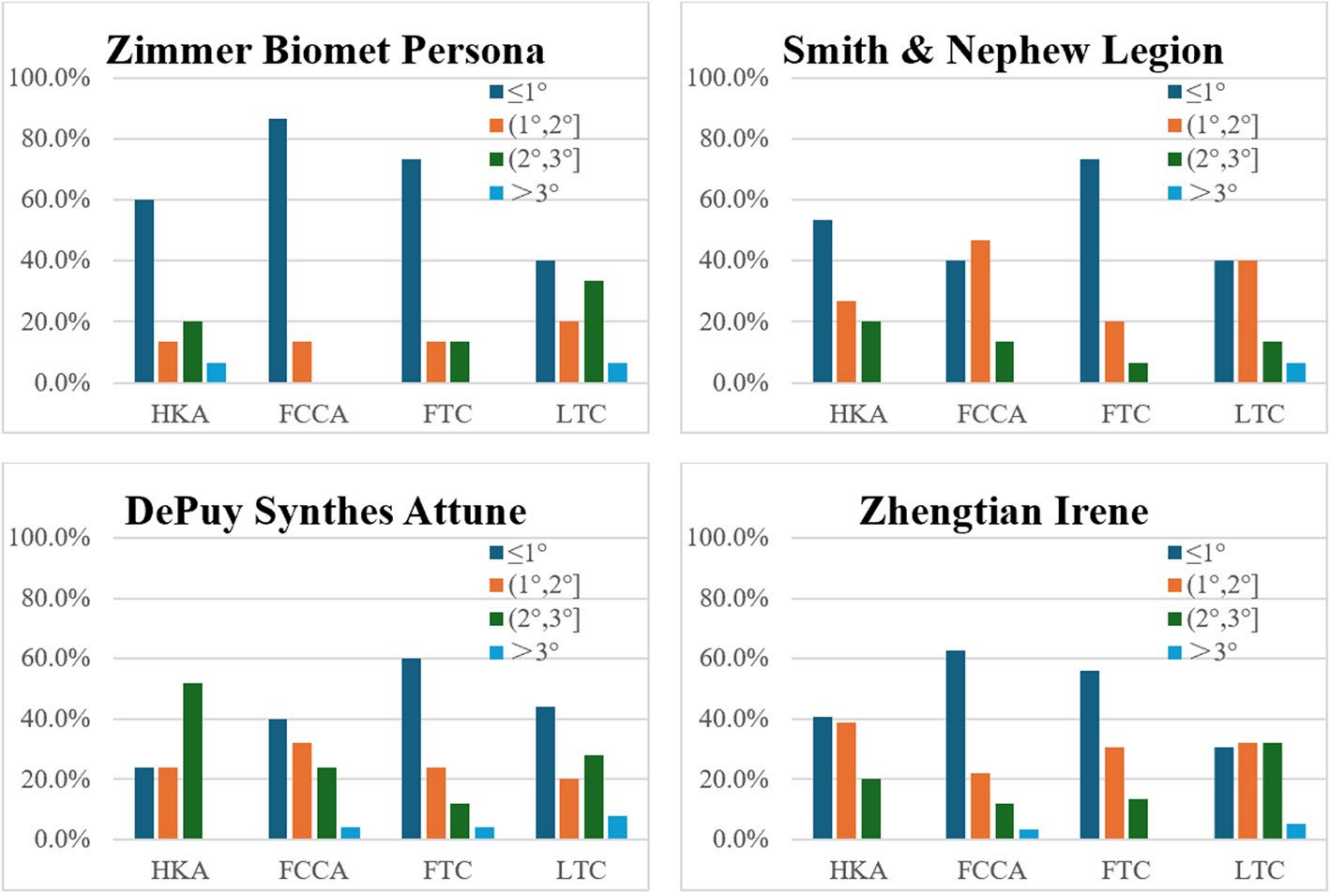
Graphs showing the difference between planned and post-operative HKA, FCCA, TCCA, and PTS divided by implant types

Subgroup analyses investigated the influence of alignment strategy (functional vs. mechanical alignment) and implant type on post-operative alignment (Table 4). The FA subgroup showed a significant difference in HKA only (1.32°, *P* < 0.001), whereas the MA subgroup showed a significant difference in HKA (1.33°, *P* < 0.001) and FCCA (−0.59°, *P* < 0.001), respectively. Implant subgroup analysis showed deviation from planned HKA across all four implant types, which was statistically significant (Table 5). Other significant deviations from planned alignment were FCCA in the DePuy Synthes (*P* = 0.045) and Zhengtian Irene (*P* < 0.0001) subgroups, as well as PTS from the Zhengtian Irene subgroup (*P* = 0.004). For both subgroup analyses, the mean difference, including a 95% confidence interval, showed deviation of less than 3°, which was radiologically acceptable. Planned implant alignment with division by implant type is illustrated in Fig. 3. The percentage of deviation from planned alignment between implant types is presented in Fig. 4 and Table 6, with all groups having more than 90% of patients having less than 3° deviation.

**Table 4:** Paired *t*-test results of Functional Alignment and Mechanical Alignment subgroups

Measurement	Planned (SD)	Post-op (SD)	Difference (SD)	95%CI lower	95%CI upper	*P*-value	
Functional Alignment Group							
HKA	178.95(1.41)	177.63(1.55)	1.32 (1.29)	0.97	1.67		< 0.001
FCCA	90.37(0.87)	90.88(1.80)	− 0.51 (1.61)	− 0.95	− 0.08		0.022
TCCA	89.41(1.04)	89.24(1.44)	0.17 (1.16)	− 0.14	0.49		0.272
PTS	86.46(0.80)	86.58(2.17)	− 0.12 (1.90)	− 0.64	− 0.39		0.635
Mechanical Alignment Group							
HKA	180.00(0.00)	178.67(0.75)	1.33(0.75)	1.13	1.52		< 0.001
FCCA	90.00(0.00)	90.59(1.21)	− 0.59(1.21)	− 0.90	− 0.27		< 0.001
TCCA	90.00(0.00)	89.80(1.24)	0.20(1.24)	− 0.12	0.52		0.219
PTS	87.00(0.00)	87.69(1.77)	− 0.69(1.78)	− 1.16	− 0.23		0.004

**Table 5:** Subgroup statistical analysis of post-operative results

	Indicators	HKA	FCCA	TCCA	PTS
Zimmer Persona	Planned (SD)	178.37(1.97)	90.33(0.98)	88.70(1.58)	85.67(0.98)
	Post-operative (SD)	177.41(1.70)	90.40(1.05)	88.47(1.57)	85.58(2.18)
	Mean Difference (SD) (Plan-post)	0.95(1.27)	− 0.06(0.83)	0.23(1.03)	0.09(1.83)
	95%CI lower	0.25	− 0.52	− 0.34	− 0.93
	95%CI upper	1.66	0.39	0.80	1.10
	T	2.918	− 0.297	0.86	0.182
	P	0.011	0.771	0.404	0.858
DePuy Synthes Attune	Planned (SD)	179.26(1.03)	90.34(0.94)	89.80(0.50)	86.80(0.47)
	Post-operative (SD)	177.47(1.45)	91.20(2.25)	89.63(1.40)	87.14(2.04)
	Mean Difference (SD) (Plan-post)	1.79(1.19)	− 0.86(2.02)	0.17(1.36)	− 0.33(2.00)
	95%CI lower	1.30	− 1.69	− 0.39	− 1.16
	95%CI upper	2.28	− 0.02	0.74	0.49
	T	7.516	− 2.116	0.635	− 0.830
	P	< 0.0001	0.045	0.531	0.415
Smith & Nephew Legion	Planned (SD)	179.01(1.18)	90.45(0.65)	89.49(0.67)	86.68(0.46)
	Post-operative (SD)	178.10(1.557)	90.83(1.52)	89.37(1.09)	86.66(2.14)
	Mean Difference (SD) (Plan-post)	0.91 (1.28)	− 0.38(1.36)	0.12(0.96)	0.02(1.90)
	95%CI lower	0.19	− 1.13	− 0.41	− 1.03
	95%CI upper	1.62	0.37	0.65	1.07
	T	2.73	− 1.081	0.478	0.035
	P	0.016	0.298	0.64	0.972
Zhengtian Irene	Planned (SD)	180.00(0.00)	90.00(0.00)	90.00(0.00)	87.00(0.00)
	Post-operative (SD)	178.67(0.75)	90.59(1.21)	89.80(1.24)	87.69(1.77)
	Mean Difference (SD) (Plan-post)	1.33(0.75)	− 0.59(1.21)	0.20(1.24)	− 0.69(1.77)
	95%CI lower	1.13	− 0.9	− 0.12	− 1.16
	95%CI upper	1.52	− 0.27	0.52	− 0.23
	T	13.504	− 3.708	1.243	− 3.002
	P	< 0.0001	< 0.0001	0.219	0.004

**Table 6:** Subgroup analysis post-operative percentage of patients with deviation < 3° of HKA, FCCA, TCCA, and PTS

Deviation < 3°	HKA	FCCA	TCCA	PTS
Zimmer Biomet Persona	93.3%	100.0%	100.0%	92.3%
DePuy Synthes Attune	100.0%	96.0%	96.0%	92.0%
Smith & Nephew Legion	100.0%	100.0%	100.0%	93.3%
Zhengtian Irene	100.0%	96.6%	100.0%	94.9%

## Discussion

Despite the proven success of TKA in alleviating pain and restoring function, patient dissatisfaction remains a persistent challenge, with malalignment being a significant modifiable risk factor [4]. Robotic-assisted TKA has emerged as a promising solution, with studies demonstrating superior implant positioning accuracy compared to conventional manual techniques [11, 20, 21]. However, the majority of existing robotic systems operate on a closed-platform model, restricting implant choice and potentially limiting their applicability in diverse patient populations. This study is, to our knowledge, the first to evaluate the radiological outcomes of an open-platform robotic TKA system, demonstrating excellent correlation between preoperative planning and postoperative alignment across four different implant designs.

Our findings indicate that the KUNWU system achieved a mean deviation of < 1.32° in hip-knee-ankle angle (HKA) and < 0.55° in femoral and tibial component alignment, well within the clinically acceptable 3° threshold associated with long-term implant survivorship [7]. Notably, while statistically significant differences were observed between planned and postoperative FCCA, TCCA, and PTS, the absolute deviations remained below 1.5°, suggesting that the system maintains high precision despite its open-platform nature.

Our study showed good interobserver reliability (Cronbach’s α > 0.873, ICC > 0.776), minimizing measurement bias in radiographic assessment. Additionally, subgroup analysis between four different implant systems under a single robotic platform also showed excellent accuracy, which supports the generalizability of our findings.

However, several limitations must be acknowledged. Firstly, a lack of a control group (manual or closed-platform robotic TKA) prevents direct comparison of alignment accuracy. Additionally, unequal group sizes across implant types restrict robust subgroup analysis. Short-term follow-up limits the assessment of long-term implant survivorship and functional outcomes.

Open-platform robotic TKA offers greater implant selection flexibility, which may be particularly beneficial in patients with atypical anatomy or those requiring customized alignment strategies (e.g., functional alignment techniques). By accommodating a wider range of implant designs, open-platform systems may also facilitate more personalized kinematic restoration, potentially improving joint stability and intraoperative soft-tissue balancing. Furthermore, the ability to integrate multiple implant systems within a single robotic workflow enhances surgical adaptability, supporting broader clinical adoption and reducing institutional reliance on proprietary technology. However, the trade-off between flexibility and implant-specific optimization warrants further investigation, particularly regarding long-term wear patterns and patient-reported outcomes. Future studies should evaluate whether specific alignment targets, enabled by robotic precision, translate into superior PROMs, functional gains (e.g., gait analysis), or long-term survivorship.

## Conclusion

The Yuanhua KUNWU open-platform robotic system demonstrated excellent radiological accuracy across three different TKA implants, with mean alignment deviations within 1.5° of preoperative plans. These findings suggest that open-platform robotics can provide surgeons with implant versatility without compromising precision. Further studies with longer follow-up, functional outcomes, and comparative controls are needed to validate its clinical superiority over conventional and closed-platform robotic techniques.

## Data Availability

No datasets were generated or analysed during the current study.

## Abbreviations

TKA: Total knee arthroplasty
HKA: Hip-knee-ankle
FCCA: Femoral component coronal alignment
TCCA: Tibial component coronal alignment
PTS: Posterior tibial slope
ICC: Interclass correlation co-efficient
PROM: Patient-reported outcome measure
FA: Functional alignment
MA: Mechanical alignment
SD: Standard deviation
CI: Confidence interval

## References

[1] Gao J, Xing D, Dong S, Lin J. The primary total knee arthroplasty: a global analysis. J Orthop Surg Res. 2020;15(1):190. 10.1186/s13018-020-01707-5. 32456654 10.1186/s13018-020-01707-5 PMC7249396

[2] Bourne RB, Chesworth BM, Davis AM, Mahomed NN, Charron KD. Patient satisfaction after total knee arthroplasty: who is satisfied and who is not? Clin Orthop Relat Res. 2010;468(1):57–63. 10.1007/s11999-009-1119-9. 19844772 10.1007/s11999-009-1119-9 PMC2795819

[3] Bierke S, Häner M, Karpinski K, Hees T, Petersen W. Midterm effect of mental factors on pain, function, and patient satisfaction 5 years after uncomplicated total knee arthroplasty. J Arthroplasty. 2020;35(1):105–11. 10.1016/j.arth.2019.08.008. 31477540 10.1016/j.arth.2019.08.008

[4] Kazarian GS, Haddad FS, Donaldson MJ, Wignadasan W, Nunley RM, Barrack RL. Implant malalignment may be a risk factor for poor patient-reported outcomes measures following total knee arthroplasty. J Arthroplasty. 2022;37(6 suppl):S129–33. 10.1016/j.arth.2022.02.087. 35248754 10.1016/j.arth.2022.02.087

[5] Lee BS, Cho HI, Bin SI, Kim JM, Jo BK. Femoral component varus malposition is associated with tibial aseptic loosening after TKA. Clin Orthop Relat Res. 2018;476(2):400–7. 10.1007/s11999.0000000000000012. 29389790 10.1007/s11999.0000000000000012 PMC6259714

[6] Kazarian GS, Lieberman EG, Hansen EJ, Nunley RM, Barrack RL. Clinical impact of component placement in manually instrumented total knee arthroplasty. Bone Joint J. 2021;103-B(9):1449–56. 10.1302/0301-620X.103B9.BJJ-2020-1639.R2. 34465158 10.1302/0301-620X.103B9.BJJ-2020-1639.R2

[7] Jeffery R, Morris R, Denham R. Coronal alignment after total knee replacement. J Bone Joint Surg Br. 1991;73(B(5)):709–14. 10.1302/0301-620X.73B5.1894655. 1894655 10.1302/0301-620X.73B5.1894655

[8] Abdel MP, Ollivier M, Parratte S, Trousdale RT, Berry DJ, Pagnano MW. Effect of postoperative mechanical axis alignment on survival and functional outcomes of modern total knee arthroplasties with cement: a concise follow-up at 20 years. J Bone Joint Surg Am. 2018;100(6):472–8. 10.2106/JBJS.16.01587. 29557863 10.2106/JBJS.16.01587

[9] Siebert W, Mai S, Kober R, Heeckt PF. Technique and first clinical results of robot-assisted total knee replacement. Knee. 2002;9(3):173–80. 10.1016/S0968-0160(02)00015-3. 12126674 10.1016/s0968-0160(02)00015-7

[10] Zhang J, Ndou WS, Ng N, et al. Robotic arm-assisted total knee arthroplasty is associated with improved accuracy and patient-reported outcomes: a systematic review and meta-analysis. Knee Surg Sports Traumatol Arthrosc. 2022;30:2696–7. 10.1007/s00167-021-06522-x. 33547914 10.1007/s00167-021-06464-4 PMC9309123

[11] Deckey DG, Rosenow CS, Verhey JT, et al. Robotic-assisted total knee arthroplasty improves accuracy and precision compared to conventional techniques. Bone Joint J. 2021;103-B(6 suppl A):74–80. 10.1302/0301-620X.103B6.BJJ-2020-2003.R1. 34053292 10.1302/0301-620X.103B6.BJJ-2020-2003.R1

[12] Tran JY, Tang AY, Wong CK, et al. Handheld imageless robotic total knee arthroplasty improves accuracy and early clinical outcomes when compared with navigation. Arthroplasty. 2025;7(1):18. 10.1186/s42836-025-00303-4. 40181427 10.1186/s42836-025-00303-4 PMC11969756

[13] Clark TC, Schmidt FH. Robot-assisted navigation versus computer-assisted navigation in primary total knee arthroplasty: efficiency and accuracy. ISRN Orthop. 2013;2013:794827. 10.1155/2013/794827. 24967115 10.1155/2013/794827 PMC4045350

[14] St Mart JP, Goh EL. The current state of robotics in total knee arthroplasty. EFORT Open Rev. 2021;6(4):270–9. 10.1302/2058-5241.6.200052. 34040804 10.1302/2058-5241.6.200052 PMC8142057

[15] Sousa PL, Sculco PK, Mayman DJ, et al. Robots in the operating room during hip and knee arthroplasty. Curr Rev Musculoskelet Med. 2020;13(3):309–17. 10.1007/s12178-020-09625-z. 32367430 10.1007/s12178-020-09625-z PMC7251009

[16] Bautista M, Manrique J, Hozack WJ. Robotics in total knee arthroplasty. J Knee Surg. 2019;32(7):600–6. 10.1055/s-0039-1681053. 30822790 10.1055/s-0039-1681053

[17] Jacofsky DJ, Allen M. Robotics in arthroplasty: a comprehensive review. J Arthroplasty. 2016;31(10):2353–63. 10.1016/j.arth.2016.05.026. 27325369 10.1016/j.arth.2016.05.026

[18] Okoro T, Tomescu S, Paterson JM, Ravi B. Analysis of the relationship between surgeon procedure volume and complications after total knee arthroplasty using a propensity-matched cohort study. BMJ Surgery, Interventions, & Health Technologies. 2021;3(1):e000072. 10.1136/bmjsit-2020-000072. (**2021 Apr 2**). 10.1136/bmjsit-2020-000072 PMC8647593 35051253

[19] Patel K, Judd H, Harm RG, Nolan JR, Hummel M, Spanyer J. Robotic-assisted total knee arthroplasty: is there a maximum level of efficiency for the operating surgeon? J Orthop. 2022;31:13–6. 10.1016/j.jor.2022.02.015. (**2022 Feb 15**). 35310516 10.1016/j.jor.2022.02.015 PMC8927899

[20] Moon YW, Ha CW, Do KH, et al. Comparison of robot-assisted and conventional total knee arthroplasty: a controlled cadaver study using multiparameter quantitative three-dimensional CT assessment of alignment. Comput Aided Surg. 2012;17(2):86–95. 10.3109/10929088.2012.654408. 22348661 10.3109/10929088.2012.654408

[21] Bellemans J, Vandenneucker H, Vanlauwe J. Robot-assisted total knee arthroplasty. Clin Orthop Relat Res. 2007;464:111–6. 10.1097/BLO.0b013e318126c0c0. 17563698 10.1097/BLO.0b013e318126c0c0

